# REG4 is a transcriptional target of GATA6 and is essential for colorectal tumorigenesis

**DOI:** 10.1038/srep14291

**Published:** 2015-09-21

**Authors:** Yoshihiro Kawasaki, Kosuke Matsumura, Masaya Miyamoto, Shinnosuke Tsuji, Masumi Okuno, Sakiko Suda, Masaya Hiyoshi, Joji Kitayama, Tetsu Akiyama

**Affiliations:** 1 Laboratory of Molecular and Genetic Information, Institute of Molecular and Cellular Biosciences, The University of Tokyo, 1-1-1, Yayoi, Bunkyo-ku, Tokyo, 113-0032, Japan; 2 Department of Surgical Oncology, Graduate school of Medicine, The University of Tokyo, 7-3-1, Hongo, Bunkyo-ku, Tokyo, 113-8655, Japan

## Abstract

The transcription factor GATA6 is a critical regulator of cell proliferation and development in the gastrointestinal tract. We have recently reported that GATA6 induces the expression of the intestinal stem cell marker LGR5 and enhances the clonogenicity and tumorigenicity of colon cancer cells, but not the growth of these cells cultured under adherent conditions. Here we show that REG4, a member of the regenerating islet-derived (REG) family, is also a target of GATA6. We further demonstrate that REG4 is downregulated by overexpression of miR-363, which suppresses GATA6 expression. Moreover, we show that GATA6-mediated activation of REG4 enhances the growth of colon cancer cells under adherent conditions and is required for their tumorigenicity. Taken together, our findings demonstrate that GATA6 simultaneously induces the expression of genes essential for the growth of colon cancer cells under adherent conditions (REG4) and genes required for their clonogenicity (LGR5), and that the miR-363-GATA6-REG4/LGR5 signaling cascade promotes the tumorigenicity of colon cancer cells.

The GATA family, a group of evolutionarily conserved zinc finger-containing transcription factors, is essential for proliferation, differentiation and development in many organs^1^,^2^. Six members of the GATA family have been identified in vertebrate species and can be classified into two subgroups based on their expression patterns and functions. GATA1, 2, and 3 are preferentially expressed in hematopoietic cells and regulate their proliferation and differentiation during hematopoiesis. GATA4, 5, and 6 are expressed in the developing cardiovascular system and in multiple tissues including the lung, liver and gastrointestinal tract^3^. In particular, GATA6 is expressed throughout the gastrointestinal epithelium, and expression is particularly high in the proliferative crypt compartment. Previous studies have revealed that deletion of *Gata6* in the intestine results in impaired crypt cell proliferation, crypt-to-surface epithelial migration, lineage maturation and gene expression in the mature mouse colonic epithelium^4^,^5^,^6^,^7^,^8^.

It has been shown that GATA6 is essential for the tumorigenicity of colorectal cancer. In our previous study, we observed that suppression of GATA6 expression inhibits the growth and tumorigenicity of colon cancer cells in a nude mouse xenograft model^9^. Furthermore, it has been reported that *Gata6* deletion in an *Apc*-null background, a mouse model of intestinal tumorigenesis, leads to prolonged survival, lower intestinal tumor burden and a decrease in the self-renewal capacity of colon adenoma stem cells^10^. It has also been shown that GATA6 plays important roles in colon cancer cell invasion and the malignant progression of Barrett's esophagus^11^,^12^. Systemic ablation of GATA6 in mice results in an embryonic lethal phenotype due to impaired endoderm differentiation^13^,^14^,^15^.

Leucine-rich repeat containing G-protein-coupled receptor 5 (LGR5), a member of the G-protein-coupled receptor (GPCR) family of proteins, is a target of Wnt signaling and marks stem cell populations in various organs, including small intestine and colon^16^. It has been shown that LGR5 is an R-spondins receptor and is essential for R-spondin-induced Wnt signaling^17^,^18^,^19^. Moreover, we have recently reported that LGR5 is a transcriptional target of GATA6 and plays important roles in the clonogenicity and tumorigenicity of colon cancer cells, but does not affect their proliferation under adherent conditions^9^. Since GATA6 is critical for both clonogenicity and proliferation under adherent conditions, this finding suggests that other genes may mediate the ability of GATA6 to promote proliferation under adherent conditions. We further have previously shown that miR-363 expression is reduced in colon tumors and that its downregulation results in an increase in GATA6 expression, which thereby enhances LGR5 expression.

The regenerating gene (REG) family members, REG1∼4, are small secreted lectin-like proteins involved in hepatic, pancreatic, gastric and intestinal cell proliferation and differentiation^20^. It has been reported that aberrant expression of REG4 is associated with the growth, survival, adhesion and resistance to apoptosis of tumor cells^21^,^22^,^23^,^24^,^25^,^26^. Furthermore, *REG4* has been identified as one of the genes up-regulated in gastric cancer-initiating cells and is thus used as a novel marker for these cells^27^.

In this study, we show that *REG4* is a target of GATA6 and that miR-363 represses REG4 transcription via its suppression of GATA6. Furthermore, we demonstrate that GATA6-mediated induction of REG4 enhances the growth of colon cancer cells under adherent conditions. Our findings suggest that GATA6 simultaneously induces the expression of genes essential for colon cancer cell growth under adherent conditions (REG4) and genes that promote their clonogenicity (LGR5). These results further support the important role of the miR-363-GATA6-REG4/LGR5 signaling cascade in the tumorigenicity of colon cancer cells.

## Results

### REG4 promotes colorectal tumorigenesis

**Figure 1:**
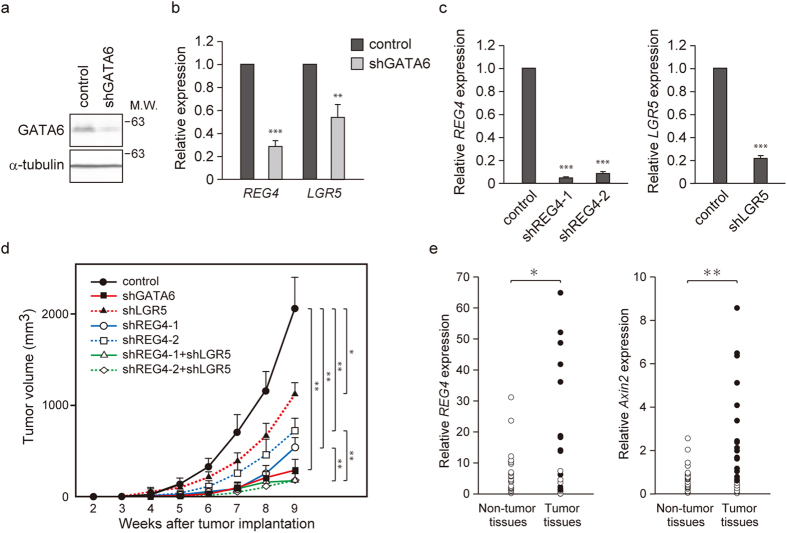
Cooperation between REG4 and LGR5 in colorectal tumorigenesis. (**a**) HT29 cells were infected with a lentivirus encoding an shRNA targeting GATA6, and lysates were subjected to immunoblotting analysis with anti-GATA6 antibody. α-tubulin served as a loading control. (**b**) qRT-PCR quantitation of *REG4* and *LGR5* was performed using total RNA from HT29 cells infected with a lentivirus encoding an shRNA targeting GATA6. Values represent the mean ± s.e.m. (n = 4). *Actin* served as an internal control. **P < 0.01, ***P < 0.001. (**c**) qRT-PCR quantitation of *REG4* and *LGR5* was performed using total RNA from HT29 cells infected with a lentivirus encoding an shRNA targeting REG4 or LGR5. Values represent the mean ± s.e.m. (n = 3). *Actin* served as an internal control. ***P < 0.001. (**d**) Nude mice (n = 8 per group) were injected subcutaneously with HT29 cells expressing the indicated shRNAs, and tumor formation was monitored. Values represent the mean ± s.e.m. *P < 0.05, **P < 0.01. (**e**) qRT-PCR analysis of *REG4* expression in human colon tumors and surrounding normal tissues. The amount of *REG4* and *Axin2* mRNA is shown as the percentage of the amount of *HPRT1* mRNA (n = 23 pairs). Filled circles represent tumor tissues in which the indicated mRNA is expressed at a higher level than in the corresponding non-tumor tissues. *Axin2* was used as a marker for the activation of Wnt/β-catenin signaling. *P < 0.05, **P < 0.01.

We analyzed microarray data obtained from DLD-1 colon cancer cells (GSE 32987)^9^ and found that silencing of GATA6 resulted in a >75% decrease in *REG4* expression. Consistent with this result, silencing of GATA6 using a lentivirus expressing a short hairpin RNA (shRNA) targeting GATA6 caused a significant downregulation of *REG4*, as well as *LGR5* in HT29 colon cancer cells (Fig. 1a,b). To investigate the significance of REG4 in the tumorigenesis of colon cancer, we generated HT29 cells expressing a lentiviral shRNA targeting REG4 and injected these subcutaneously into nude mice. We found that the growth of these tumor cells was inhibited compared with control lentivirus-infected cells (Fig. 1c,d). These results suggest that REG4 is essential for colorectal tumorigenesis. Since knockdown of GATA6 resulted in a more significant inhibition of tumor growth than REG4 or LGR5 knockdown alone, we examined the effect of REG4 and LGR5 double knockdown on the tumorigenicity of HT29 cells. We found that the double knockdown suppressed the growth of tumor cells in nude mice to the levels observed with the GATA6 knockdown (Fig. 1d). Similar results were obtained with the colon tumor cell line LS180 (Supplementary Fig. S1a–c). Thus, REG4 and LGR5 may contribute independently to the tumorigenicity of colon cancer cells.

We next examined REG4 expression in human colorectal tumors and adjacent non-tumor tissues by qRT-PCR analysis. As reported previously^28^,^29^, *REG4* expression was found to be elevated in colon tumor tissues compared with the surrounding non-tumor tissues (Fig. 1e). We found no significant correlation between the expression ratios (Tumor/Normal) of *REG4* and *Axin2*, a marker of Wnt/β-catenin activation, in clinical specimens.

### REG4 is a transcriptional target of GATA6 in colon cancer cells

**Figure 2:**
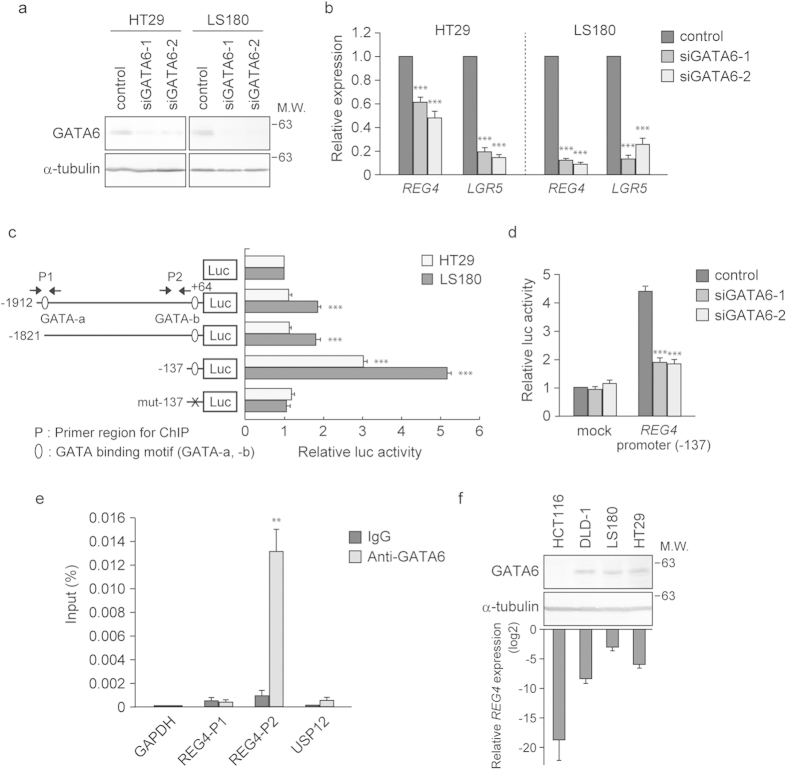
GATA6 induces the expression of REG4 in colon cancer cells. (**a**) Lysates prepared from HT29 and LS180 cells transfected with siRNA targeting GATA6 were subjected to immunoblotting analysis with anti-GATA6 antibody. α-tubulin served as a loading control. (**b**) qRT-PCR analysis of *REG4* and *LGR5* was performed using total RNA from HT29 and LS180 cells transfected with siRNA against GATA6. *GAPDH* served as an internal control. Values represent the mean ± s.e.m. (n = 6). ***P < 0.001. (**c**) (Left) Luciferase (Luc) reporter constructs containing the fragments of the *REG4* promoter region. Potential GATA-binding motifs (A/TGATAA/G) are represented by ellipses and primer pairs used in ChIP assays are indicated as P1 and P2, respectively. The mutated elements are shown with the cross mark. (Right) HT29 and LS180 cells were transfected with the indicated promoter constructs and luciferase activity was measured. Values represent the mean ± s.d. (n = 3). ***P < 0.001. (**d**) Luciferase assays were performed using LS180 cells that had been transfected with GATA6 or control siRNA and then with the REG4 reporter construct. Values represent the mean ± s.d. (n = 4). ***P < 0.001. (**e**) ChIP analysis of the *REG4* promoter in LS180 cells using antibody against GATA6 or rabbit IgG. The positions of primers used for PCR are indicated by arrows in Fig. 2c. *GAPDH* and *USP12* promoter regions served as negative and positive controls, respectively. Values represent the mean ± s.e.m. (n = 3). **P < 0.01. (**f**) Comparison of GATA6 and REG4 expression in colon cancer cell lines by immunoblotting (upper) and qRT-PCR (lower) analyses. Lysates prepared from the indicated cells were analysed by immunoblotting with antibody against GATA6. α-tubulin served as a loading control. *GAPDH* served as an internal control. Values represent the mean ± s.d. (n = 2).

We investigated whether transcription of *REG4* is directly regulated by GATA6. We found that silencing of GATA6 led to the downregulation of both *REG4* and *LGR5* in HT29 and LS180 cells (Fig. 2a,b). To investigate the molecular mechanisms by which GATA6 upregulates REG4 expression, we examined the activities of various REG4 promoter fragments fused to the luciferase gene (−1912/Luc, −1821/Luc and −137/Luc in Fig. 2c). There are two GATA-binding sites located 2 and 1840 bp upstream of the transcription start site of the *REG4* gene. When transfected into HT29 or LS180 cells, the luciferase activity of the -137/Luc reporter was significantly enhanced compared to that of the control reporter (Fig. 2c). Furthermore, knockdown of GATA6 markedly reduced the activity of −137/Luc in LS180 cells (Fig. 2d). In addition, we measured the activity of a reporter construct in which the GATA-binding motif, AGATAA (GATA-b in Fig. 2c), had been replaced with ACCCCA (mut-137/Luc in Fig. 2c). The activity of mut-137/Luc was markedly reduced in HT29 and LS180 cells, suggesting that GATA6 upregulates *REG4* expression by binding to the GATA-b site in the *REG4* promoter region. We also found that deletion of the region between -1812 and -137 caused a significant increase in luciferase activity. This result suggests that a negative regulatory element(s) is present in this region of the *REG4* promoter. In addition, the activities of the -1912/Luc and −1821/Luc reporters were significantly elevated in LS180 cells but not in HT29 cells, suggesting that an LS180 cell type-specific enhancer element resides within the −1821 to +64 region.

To determine whether endogenous GATA6 binds to the *REG4* promoter region *in vivo*, we performed chromatin immunoprecipitation (ChIP) assays on LS180 and HT29 cell lysates using antibody against GATA6 or control rabbit IgG. We found significant enrichment of a DNA fragment containing the GATA-b site from chromatin precipitated with anti-GATA6 antibody (Fig. 2e and Supplementary Fig. S2). Our findings imply that GATA6 directly induces the expression of REG4 through the GATA-binding motif present in its promoter region.

We subsequently examined the expression levels of GATA6 protein and *REG4* mRNA in colon cancer cell lines by immunoblotting and qRT-PCR analyses, respectively. We could readily detect GATA6 protein and *REG4* mRNA in DLD-1, LS180 and HT29 cells (Fig. 2f). By contrast, we could not detect either GATA6 protein or *REG4* mRNA in HCT116 cells. These results seem to be consistent with the view that REG4 is a transcriptional target gene of *GATA6*.

### The GATA6-REG4 pathway is essential for the growth of colon cancer cells

**Figure 3:**
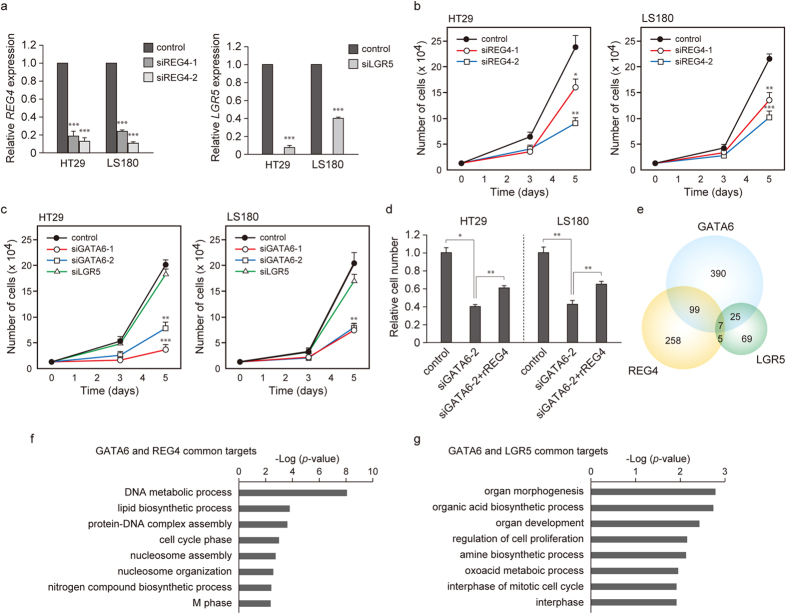
GATA6-mediated upregulation of REG4 is essential for proliferation of colon cancer cells under adherent conditions. (**a**) qRT-PCR analysis of *REG4* and *LGR5* was performed using total RNA from HT29 and LS180 cells transfected with REG4- or LGR5-specific or control siRNAs. *GAPDH* served as an internal control. Values represent the mean ± s.e.m. (n = 3). ***P < 0.001. (**b**,**c**) Effects of REG4 (**b**), GATA6 or LGR5 (**c**) knockdown on the growth of HT29 (left) and LS180 (right) cells cultured under adherent conditions. Cells transfected with the indicated siRNAs were plated at the density of 1.25 × 10^4^ cells into 48-well plates and cell numbers were counted after 3 and 5 days. Values represent the mean ± s.e.m. (n = 3). *P < 0.05, **P < 0.01, ***P < 0.001. (**d**) Treatment with recombinant human REG4 (rREG4) restores the growth of GATA6-knockdown HT29 and LS180 cells. Recombinant REG4 was added to the culture medium and cell counts were performed after 7 days of culture. Values represent the mean ± s.e.m. (n = 3). (**e**) Microarray analysis of genes regulated by GATA6, REG4 or LGR5 in HT29 cells. Venn diagram of genes downregulated by GATA6 (blue), REG4 (orange) or LGR5 (green) knockdown. The significance of the overlap was calculated by hypergeometric distribution (P = 0). The number of genes within each category is indicated. (**f**) GO analysis of the genes shared by the GATA6 and REG4 signatures indicated in (**e**). Significance is expressed as GO enrichment scores (-Log P-value). (**g**) GO analysis of the genes shared by the GATA6 and LGR5 signatures indicated in (**e**).

We have recently reported that knockdown of LGR5, in contrast to knockdown of GATA6, does not significantly affect the growth of colon cancer cells cultured under adherent conditions^9^. We therefore examined the effects of REG4 knockdown on the growth of HT29 and LS180 cells under adherent conditions. We found that knockdown of REG4 expression by siRNA inhibited the growth of these cell lines (Fig. 3a,b). We also ascertained that silencing of GATA6, unlike LGR5, led to marked growth inhibition (Fig. 3a,c). Furthermore, the addition of recombinant REG4 protein to the culture medium partially restored the growth of GATA6 knockdown cells (Fig. 3d). These observations suggest that the GATA6-REG4 pathway, but not the GATA6-LGR5 pathway, is essential for the growth of colon cancer cells under adherent conditions.

We next examined gene expression in HT29 cells in which GATA6, REG4 or LGR5 had been knocked down. DNA microarray analysis showed that the expression profiles of REG4-knockdown cells and LGR5-knockdown cells partially overlapped with that of GATA6-knockdown cells (Fig. 3e), consistent with the notion that both *REG4* and *LGR5* are transcriptional target genes of GATA6. Nonetheless, gene ontology (GO) analysis indicated that the common targets of GATA6 and REG4 are enriched for genes involved in “DNA metabolic processes”, “protein-DNA complex assembly” and “cell cycle phase” (Fig. 3f), whereas those of GATA6 and LGR5 are enriched for genes involved in “organ morphogenesis”, “organ development” and “regulation of cell proliferation” (Fig. 3g). In addition, the limited overlap between the REG4 and LGR5 knockdown datasets (Fig. 3e) appears to be consistent with the notion that REG4 and LGR5 function independently downstream of GATA6.

### miR-363 represses the growth of colon cancer cells by suppressing the GATA6-REG4 pathway

**Figure 4:**
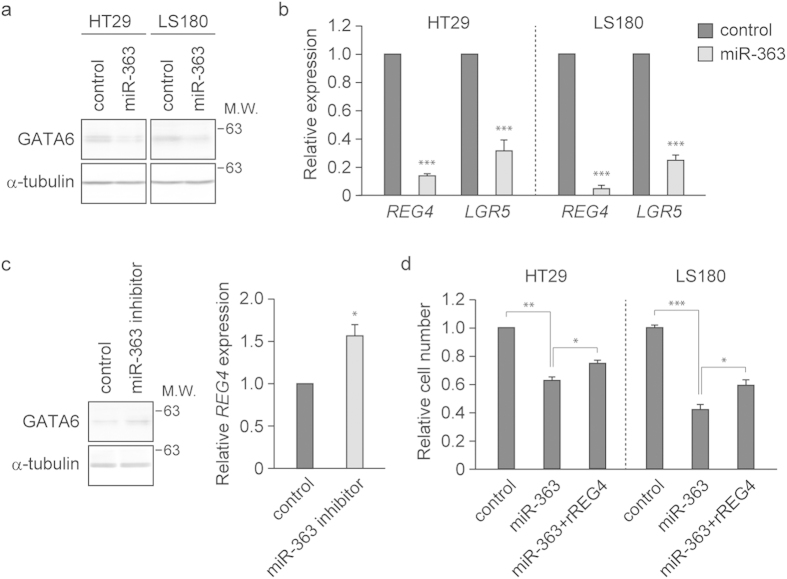
miR-363 inhibits the growth of colon cancer cells by suppressing the GATA6-REG4 pathway. (**a**,**b**) Lysates from HT29 and LS180 cells transfected with pre-miR-363 or control miRNA were subjected to immunoblotting analysis with anti-GATA6 antibody (**a**). qRT-PCR analysis of *REG4* and *LGR5* was performed using total RNA from these cells (**b**). α-tubulin served as a loading control. *GAPDH* served as an internal control. Values represent the mean ± s.e.m. (n = 4). ***P < 0.001. (**c**) SW403 cells were treated with antisense oligonucleotide targeting miR-363, followed by immunoblotting analysis with anti-GATA6 antibody (left) or qRT-PCR analysis of *REG4* (right). Values represent the mean ± s.e.m. (n = 3). *P < 0.05. (**d**) Treatment with recombinant REG4 restores the growth of HT29 and LS180 cells infected with miR-363 expressing lentivirus. Human recombinant REG4 was added to the culture medium and the number of cells was counted after 7 days of culture. Values represent the mean ± s.e.m. (n = 3).

We have recently shown that miR-363 suppresses the expression of GATA6 by directly targeting the 3′-UTR of *GATA6* mRNA^9^. In agreement with our previous observation, exogenous miR-363, unlike the miR-control, led to reductions in the expression of GATA6 in HT29 and LS180 cells (Fig. 4a). We also found that exogenously expressed miR-363 markedly repressed the expression of *REG4* as well as *LGR5* (Fig. 4b). Furthermore, overexpression of antisense oligonucleotide targeting miR-363 resulted in enhanced expression of GATA6 and *REG4* mRNA in SW403 cells, which express miR-363 at high levels (Fig. 4c). In addition, we could not identify any putative miR-363 target sites within the 3′-UTR of *REG4* mRNA by *in silico* analysis (TargetScan). These findings suggest that miR-363 downregulates REG4 expression via inhibition of GATA6 expression in colon cancer cells.

To examine whether suppression of REG4 by miR-363 could cause decreased growth of colon cancer cells under adherent conditions, HT29 and LS180 cells were infected with a lentivirus carrying miR-363. We found that miR-363-expressing cells had reduced growth relative to control lentivirus-treated cells (Fig. 4d). Moreover, the addition of recombinant REG4 protein to the culture medium partially restored the growth of cells expressing miR-363 (Fig. 4d). Thus, miR-363 may repress the growth of colon cancer cells via inhibition of REG4 expression.

It has been shown that promoter methylation is responsible for the silencing of miR-363 in head and neck squamous cell carcinoma^30^. We have therefore examined the effects of 5-azacytidine, an inhibitor of DNA methyltransferase, on the expression of miR-363 in colon cancer cells. We have found that treatment with 5-azacytidine resulted in an increase in miR-363 expression in LS180 and HT29 cells (Supplementary Fig. S3). Thus, promoter methylation may play an important role in the suppression of miR-363 expression.

## Discussion

We and others have recently reported that GATA6 directly induces the expression of *LGR5* through the GATA-binding motif present in its promoter region in colon cancer cells^9^,^10^. Suppression of GATA6 or LGR5 expression attenuates the tumorigenicity of colon cancer cells in nude mice, whereas decreased expression of LGR5, but not GATA6, does not significantly affect the growth of these cells cultured under adherent conditions. Furthermore, GATA6 suppression has more significant effects on tumorigenicity than LGR5 suppression. In addition, exogenous expression of LGR5 only partially rescues the tumorigenicity of colon cancer cells in which GATA6 was knocked down. These findings imply that GATA6 has other critical target genes in addition to LGR5. To obtain new insights into the role of GATA6 in proliferation and tumorigenesis, we attempted to identify GATA6 target genes responsible for cell growth.

**Figure 5:**
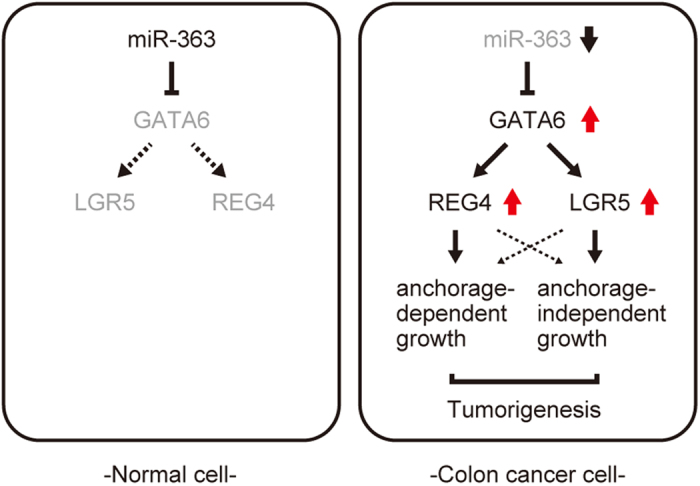
Model for GATA6-mediated colorectal tumorigenesis. The expression of GATA6 is enhanced in colon cancer cells because miR-363 is downregulated. GATA6 simultaneously activates the transcription of genes required for growth (REG4) and clonogenicity (LGR5), and cooperation between the GATA6/REG4 and GATA6/LGR5 pathways may be important for the tumorigenicity of colon cancer cells.

In the present study, we identified *REG4* as a novel target gene of GATA6 and showed that GATA6-mediated activation of REG4 expression is essential for the growth of colon cancer cells under adherent conditions in culture and their tumorigenicity *in vivo*. Furthermore, we found while individual knockdown of either REG4 or LGR5 partially inhibited tumor cell growth in nude mice, the double REG4/LGR5 knockdown inhibited tumor growth to an extent similar to the GATA6 knockdown. Consistent with this, there is only a small overlap between the gene expression profiles of REG4- and LGR5-knockdown cells. Thus, REG4 and LGR5 may function independent of each other to mediate GATA6-induced tumorigenesis (Fig. 5). In line with our previous findings, GATA6 has been shown to drive the expression of LGR5 in colon adenoma stem cells^10^. It has also been reported that coordinated expression of REG4 and aldehyde dehydrogenase 1 (ALDH1) enhances the tumorigenicity of diffuse-type gastric carcinoma-initiating cells^27^. It would therefore be intriguing to assess the role of the GATA6/REG4 and GATA6/LGR5 pathways in the properties of colon cancer stem cells.

We showed that overexpression of miR-363 downregulates REG4, and that this is via suppression of GATA6 expression. Furthermore, we demonstrated that the addition of recombinant REG4 to the culture medium partially restores the proliferation of colon cancer cells expressing miR-363 or those in which GATA6 was knocked down. Thus, reduced miR-363 expression in colon cancer cells may contribute to the upregulation of GATA6 and consequently of REG4 (Fig. 5). Since the effect of recombinant REG4 was partial, we speculate that GATA6 may have other targets that play roles in cell growth, at least under adherent culture conditions. In addition, miR-363 is known to target many genes in addition to GATA6, including *S1PR1*, which is crucial for the growth of hepatocellular carcinoma cells^31^. It remains to be investigated whether other targets such as *S1PR1* may be involved in colorectal tumorigenesis.

As reported previously^28^,^29^,^32^, we found that *REG4* and *LGR5* expression was elevated in many colon tumors. However, no significant negative correlation was detected between *miR-363* and *LGR5* or *REG4* expression ratios (Tumor/Normal) in clinical samples and colon cancer cell lines. Consistent with these results, it has been shown that LGR5 expression in colon cancer cells is driven by mutational activation of the β-catenin/TCF complex^16^. It has also been reported that REG4 is a direct target of the intestinal transcriptional factor CDX2^33^. Furthermore, GATA6 acts in combination with other transcriptional factors, including TCF4^10^ and CDX2^34^, to stimulate or repress gene expression. Thus, the expression levels of *LGR5* and *REG4* may not be simply determined by those of *miR-363* and GATA6.

DNA microarray analysis showed that the gene expression pattern of REG4-knockdown cells resembles that of GATA6-knockdown cells. GO analysis of the genes identified in REG4 and GATA6 knockdown cells revealed a highly significant enrichment for probe sets annotated as “cell cycle phase” (*P* = 9.7 × 10^−4^). Consistent with these results, it has been suggested that REG4 contributes to cell proliferation and anti-apoptosis by transactivating EGFR^21^. However, the physiological functions of REG4 and the signaling pathway are still poorly understood, as its receptor has not been identified. EGFR has been shown to be transactivated by a wide variety of G protein-coupled receptor (GPCR) agonists, including thrombin, lysophosphatidic acid (LPA), angiotensin II, endothelin-1 (ET-1), and carbachol. It is therefore possible that the elusive receptor for REG4 may be found within the large family of GPCRs^35^. Identification of the receptor for REG4 will be necessary to fully elucidate its functions and modes of intracellular signaling.

In conclusion, our findings demonstrate that GATA6 simultaneously induces the expression of genes essential for the growth (REG4) and clonogenicity (LGR5), and that cooperation between the GATA6/REG4 and GATA6/LGR5 pathways is important for the tumorigenicity of colon cancer cells. This notion is consistent with recent findings showing that deletion of GATA6 suppresses intestinal tumorigenesis in an Apc-null background^10^. Thus, the miR-363-GATA6-REG4/LGR5 signaling cascade may serve as a new therapeutic target for patients with colon cancer. In particular, monoclonal antibodies targeting REG4 or its receptor could hold promise as novel anti-tumor reagents.

## Methods

### Cell culture and antibodies

HT29 cells were cultured in RPMI 1640 medium supplemented with 10% fetal bovine serum (FBS). LS180 cells were cultured in Dulbecco’s modified Eagle’s medium (DMEM) containing 10% FBS. SW403 cells were cultured in Leibovitzs L-15 medium supplemented with 10% FBS. Cells were exposed to 5-azacytidine (Sigma: LS180, 1 μM; HT29, 5 μM) for 4 days. Fresh medium containing 5-aza was replaced every 24 h. Rabbit polyclonal antibody against GATA6 was obtained from Cell Signaling Technology. Mouse monoclonal antibody specific for α-tubulin was from Calbiochem.

### Clinical materials

Human colorectal tumors and adjacent normal tissues were obtained from patients who provided informed consent and underwent surgical treatment at the University of Tokyo Hospital.

### siRNAs, miRNAs and transfection

Transient transfection of siRNA or miRNA was performed with Lipofectamine RNAiMAX (Invitrogen) according to the manufacturer’s protocol. siRNAs and miRNAs used were as follows: stealth siRNA duplexes (GATA6-1: HSS104009, GATA6-2: HSS178134 and LGR5: HSS189418, Invitrogen); silencer siRNAs (REG4-1: s38390 and REG4-2: s38392, Ambion); synthetic miRNA (Pre-miR-363, Ambion); miRNA inhibitor (mirVana miR-363 inhibitor, Ambion). Stealth siRNA negative control low GC duplex #2 (Invitrogen), silencer negative control 1 siRNA (Ambion) and Pre-miR and mirVana inhibitor negative controls (Ambion) served as negative controls.

### Immunoblotting analyses and the lentiviral expression system

Immunoblotting analyses and Lentiviral-mediated expression of shRNA or miRNA were performed as described previously^9^. shRNA targeting luciferase served as a negative control. Oligonucleotide sequences of shRNA used in this study are shown in Supplementary Table S1.

### Plasmid construction and luciferase reporter assays

The upstream and downstream regions of the *REG4* gene transcription start site were obtained by PCR amplification from human genomic DNA and subcloned into pGL4.20 [luc2/Puro] (Promega). All PCR products were amplified with KOD-Plus-Neo (TOYOBO). Cells were transfected with 1 μg of the firefly luciferase reporter and 0.1 μg of the pRL-TK vector (internal control). Luciferase activities were determined with the Dual-luciferase reporter assay kit (Promega) and a luminometer (Mithoras LB 940, BERTHOLD).

### RNA expression analysis

Total RNA from cultured cells and clinical samples were obtained using TRIsure reagent (BIOLINE) and NucleoSpin RNA II (MACHEREY-NAGEL). Reverse transcription of total RNA was performed using PrimeScript RT Master Mix (TAKARA). mRNA expression was determined by qRT-PCR using the LightCycler 480 Real-Time PCR System (Roche). The sequences of primers are shown in Supplementary Table S1.

### Chromatin-immunoprecipitation (ChIP)

We performed chromatin immunoprecipitations using anti-GATA6 antibody or Rabbit IgG (CHEMICON) following the instructions of the manufacturer (Upstate Biotech). Immunoprecipitated DNA fragments were analysed by qRT-PCR using primers directed against a region containing the predicted GATA6 binding element present in the promoter region of *REG4* or *USP12* (positive control). A region in the *GAPDH* promoter region served as a negative control. The sequences of primers are shown in Supplementary Table S1.

### Cell proliferation assays

Cells transfected with siRNAs were plated at 1.25 × 10^4^ cells per well in 48-well plates. Then, cells were harvested by trypsinization and counted after 3 and 5 days in culture. For experiments shown in Fig. 3d and 4d, cells were seeded in 24-well plate at the density of 2.5 × 10^4^ cells per well. After 24 h, the media were replaced with fresh media containing 10 nM recombinant human REG4 (rREG4; Sino Biological Inc.) and 1% fetal bovine serum. The addition of exogenous rREG4 was performed every other day and cell counts were performed 6 days after initial treatment.

### Tumorigenicity in nude mice

Cells in a 50% Matrigel (BD Biosciences) suspension were injected (HT29, 1 × 10^3^; LS180, 5 × 10^2^ cells/mouse) subcutaneously into the hind legs of nude mice. Tumor size was measured by a slide caliper and tumor volume was determined by the following formula: π/6 × (L × W^2^), where L is the length and W is the width of the tumor (length is greater than width). 6–10-week-old nude mice were used for *in vivo* studies. All animal experiments were carried out in accordance with ‘the Guidelines for Proper Conduct of Animal Experiments’ provided by the Science Council of Japan, and were approved by the Ethics Committee of the Institute of Molecular and Cellular Biosciences, The University of Tokyo.

### Gene expression profile and gene ontology analyses

Total RNAs isolated from HT29 cells were preprocessed for hybridization to Affymetrix one-color microarrays [Affymetrix Human Gene 2.0 ST Array (Affymetrix)] using the GeneChip WT PLUS Reagent Kit (Affymetrix) following the manufacturer's protocol. Evaluation of data for microarray analysis was performed using Gene Spring version 12.6.1 (Agilent Technologies). Genes that exhibited >1.4 fold decrease in expression (treated versus control) were considered as differentially regulated. GO analysis was performed with the DAVID Bioinformatics resource. The microarray data are deposited on the Gene Expression Omnibus (accession number GSE62319).

### Statistical analyses

Student’s *t*-test and Mann-Whitney *U*-test were used for statistical evaluation of differences between groups and statistical significance was defined as *P*-value < 0.05.

## Additional Information

**Accession codes:** The accession number for the microarray data is GSE62319.

**How to cite this article**: Kawasaki, Y. *et al.* REG4 is a transcriptional target of GATA6 and is essential for colorectal tumorigenesis. *Sci. Rep.* **5**, 14291; doi: 10.1038/srep14291 (2015).

## Supplementary Material

Supplementary Information
