# Supplementary material for: REG4 is a transcriptional target of GATA6 and is essential for colorectal tumorigenesis
Source: Sci Rep. 2015 Sep 21;5:14291. doi: 10.1038/srep14291 (PMC4585703; doi:10.1038/srep14291)
Supplement: Supplementary Information [file srep14291-s1.pdf]

Supplementary Information

REG4 is a transcriptional target of GATA6 and is essential for colorectal tumorigenesis

Yoshihiro Kawasaki[1*], Kosuke Matsumura[1], Masaya Miyamoto[1], Shinnosuke Tsuji[1], Masumi Okuno[1], Sakiko Suda[1], Masaya Hiyoshi[2], Joji Kitayama[2], Tetsu Akiyama[1*]

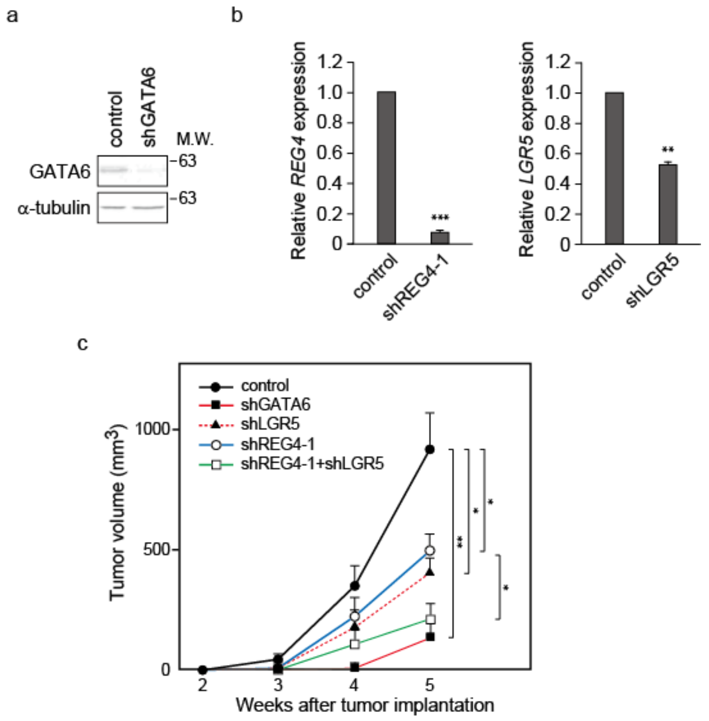

Supplementary Figure S1. Cooperation between REG4 and LGR5 is required for the tumorigenicity of LS180 cells. (a) Lysates from LS180 cells infected with a lentivirus encoding GATA6 shRNA were subjected to immunoblotting analysis with anti-GATA6 antibody. α-tubulin served as a loading control. (b) qRT-PCR analysis of *REG4* and *LGR5* was performed using total RNA from LS180 cells infected with a lentivirus encoding the indicated shRNAs. Values represent the mean ± s.e.m. (n = 3). *Actin* served as an internal control. **, P < 0.01, ***, P < 0.001. (c) Nude mice (n = 8 per group) were injected subcutaneously with LS180 cells infected with a lentivirus expressing the indicated shRNAs, and tumor formation was monitored. Values represent the mean ± s.e.m. *, P < 0.05, **, P < 0.01.

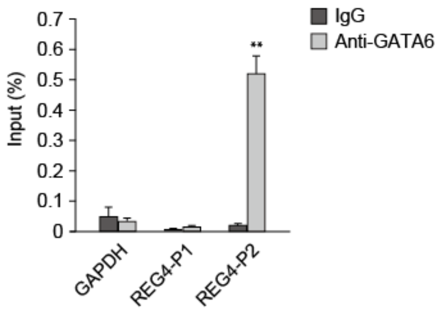

Supplementary Figure S2. ChIP analysis of the *REG4* promoter in HT29 cells using anti-GATA6 antibody. The positions of primers used for PCR are indicated by arrows in Fig. 2c. *GAPDH* promoter region served as a negative control. Values represent the mean ± s.e.m. (n = 3). **, P < 0.01.

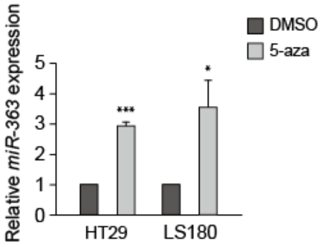

Supplementary Figure S3. Effects of 5-azacytidine on the expression of miR-363 in colon cancer cells. qRT-PCR analysis of *miR-363* was performed using total RNA from HT29 and LS180 cells treated with 5-azacytidine (5-aza) or DMSO. Values represent the mean ± s.e.m. (n = 3-4). *U6* served as an internal control. *, P < 0.05, ***, P < 0.001.

Supplementary Table S1. List of primer and shRNA target sequences.

qRT-PCR	REG4	Fw	CCTTGCACTAGCTACATCCTCA
		Rv	AAAACCATCCAGGAGCACAG
	LGR5	Fw	TCCAACCTCAGCGTCTTCAC
		Rv	CGCAAGACGTAACTCCTCCA
	Axin2	Fw	GCCAATGGCCAAGTGTCTC
		Rv	GGCTCTCCAACTCCAGCTTC
	GAPDH	Fw	GCACCGTCAAGGCTGAGAAC
		Rv	TGGTGAAGACGCCAGTGGA
	Actin	Fw	CGTCACCAACTGGGACGACA
		Rv	CTTCTCGCGGTTGGCCTTGG
	miR-363	Fw	AATTGCACGGTATCCATCTGTA

ChIP assay	REG4-P1	Fw	TGAGGATGCAAAGGCACAAG
		Rv	CCCATCATCCAAGCGGTGT
	REG4-P2	Fw	TTGCACACAGGGAGAGGTTC
		Rv	TGAATCATATAAGAGACGGCTTCAG
	GAPDH	Fw	TGCGTGCCCAGTTGAACCAG
		Rv	AACAGGAGGAGCAGAGAGCGAAGC
	USP12	Fw	TATGGCCAGTGGGAATTGAT
		Rv	GCCTCACCAACCCTCATAAA

shRNA	REG4-1	5'-GAGATGAGCTCCAATAACAAC-3'
	REG4-2	5'-GAAGCCAGCCGATATGGATTG-3'
	GATA6	5'-CTGGTAATAGCAATAATTCCATT-3'
	LGR5	5'-ATGGACGACCTTCATAAGAAAGA-3'
	luciferase	5'-GATTTCGAGTCGTCTTAATGT-3'
